# Antimicrobial Efficacy of Two Surface Barrier Discharges with Air Plasma against *In Vitro* Biofilms

**DOI:** 10.1371/journal.pone.0070462

**Published:** 2013-07-24

**Authors:** Rutger Matthes, Claudia Bender, Rabea Schlüter, Ina Koban, René Bussiahn, Stephan Reuter, Jürgen Lademann, Klaus-Dieter Weltmann, Axel Kramer

**Affiliations:** 1 Institute for Hygiene and Environmental Medicine, University Medicine Greifswald, Greifswald, Germany; 2 Institute of Microbiology, University of Greifswald, Greifswald, Germany; 3 Unit of Periodontology, Dental School, University Medicine Greifswald, Greifswald, Germany; 4 Leibniz Institute for Plasma Science and Technology (INP Greifswald), Greifswald, Germany; 5 Centre for Innovation Competence (ZIK) Plasmatis, Greifswald, Germany; 6 Centre of Experimental and Applied Cutaneous Physiology (CCP), Department of Dermatology, Venerology and Allergology, Charité – Universitätsmedizin Berlin, Berlin, Germany; The Scripps Research Institute and Sorrento Therapeutics, Inc., United States of America

## Abstract

The treatment of infected wounds is one possible therapeutic aspect of plasma medicine. Chronic wounds are often associated with microbial biofilms which limit the efficacy of antiseptics. The present study investigates two different surface barrier discharges with air plasma to compare their efficacy against microbial biofilms with chlorhexidine digluconate solution (CHX) as representative of an important antibiofilm antiseptic. *Pseudomonas aeruginosa* SG81 and *Staphylococcus epidermidis* RP62A were cultivated on polycarbonate discs. The biofilms were treated for 30, 60, 150, 300 or 600 s with plasma or for 600 s with 0.1% CHX, respectively. After treatment, biofilms were dispensed by ultrasound and the antimicrobial effects were determined as difference in the number of the colony forming units by microbial culture. A high antimicrobial efficacy on biofilms of both plasma sources in comparison to CHX treatment was shown. The efficacy differs between the used strains and plasma sources. For illustration, the biofilms were examined under a scanning electron microscope before and after treatment. Additionally, cytotoxicity was determined by the MTT (3-(4,5-Dimethylthiazol-2-yl)-2,5-diphenyltetrazolium bromide) assay with L929 mouse fibroblast cell line. The cell toxicity of the used plasma limits its applicability on human tissue to maximally 150 s. The emitted UV irradiance was measured to estimate whether UV could limit the application on human tissue at the given parameters. It was found that the UV emission is negligibly low. In conclusion, the results support the assumption that air plasma could be an option for therapy of chronic wounds.

## Introduction

Physical plasmas under atmospheric conditions, operated near room temperature, can be used to inactivate microorganisms successfully and are discussed as possible treatment method in health care [1], [2]. Their development has generated a new field of research, the so-called plasma medicine [3]. Chronic wounds, device related infections as well as inflammations of implants are often associated with microbial colonisations [4], [5]. The formation of biofilms protects the microorganism against antiseptic treatment and host defences. Additionally, the biofilms prolong the inflammation processes in chronic wounds. The efficacy of antiseptics is limited by tissue toxicity [6], [7], [8]. Additionally, chronically infected wounds increase therapy costs, they are painful and impair the patients quality of life [9]. Moreover, sometimes the wound does not heal despite correct treatment [10]. Therefore alternative treatment methods are required. The treatment of chronically infected wounds by tissue tolerable plasma (TTP) is an interesting field of investigation [11]. Investigations carried out in this respect with a TTP plasma jet [12] – the so-called kinpen 09 [13] – resulted in antibiofilm effects [14], [15], inactivation of drug resistant bacteria [16] as well as tissue activation [17] and improvement of tissue regeneration, which has meanwhile been confirmed on real wounds of humans and dogs [18], [19].

A review of different plasma sources for medical applications including skin and wound treatment, and the relevant physical and biological mechanisms has already been given by Park et al. [20].

Often, the efficacy of many different plasma sources for medical use was investigated on bacteria spread on nutrient agar plates. Those practices falsify *in vivo* conditions, because bacteria mostly live in biofilms, also in chronic wounds [21]. Investigations of antimicrobial effects on *in vitro* biofilms with different plasma sources are of high interest for potential wound treatment.

The plasma chemistry and the interaction with living systems are very complex and currently under investigation by many research groups [22], [23]. Reactive oxygen species (ROS) and reactive nitrogen species (RNS) are discussed as main effectors for antimicrobial mechanisms of plasma [24], UV radiation and pH variations seem to be supportive [25]. To enhance the understanding of interactions between plasma and microorganisms and the development of suitable plasma devices, the antimicrobial efficacy of different plasma sources on biofilms is to be investigated and compared. Surface dielectric barrier discharge (SBD) plasmas could be suitable for wound treatment because the generated plasma spreads over a large area, does not need the substrate as second electrode [3] and the physical parameters can be modified to generate a plasma with tissue tolerable properties.

In this study, the antimicrobial efficacy of two different SBD plasma sources was investigated for different exposure times on *Pseudomonas aeruginosa* and *Staphylococcus epidermidis* as biofilm forming organisms. A high antimicrobial efficacy on biofilms was expected due to the high amount of released ROS [26], [27]. *Pseudomonas aeruginosa* is ubiquitous in wet habitats and frequently identified in chronic wounds. *Staphylococcus epidermidis* is a common skin flora organism and often isolated from implants or catheter associated infections and chronic wounds [28], [29].

The results of both plasma sources were compared to the antimicrobial efficiency of chlorhexidine digluconate (CHX). CHX as an important antiseptic is regarded as gold standard for dental biofilm treatment [30] and also used in wound dressings [31]. Additionally, UV emission was measured and cytotoxicity on a fibroblasts cell line was examined to evaluate the potential applicability of the generated plasmas of both SBD plasma sources on living tissue.

## Materials and Methods

### Plasma Sources

**Figure 1:**
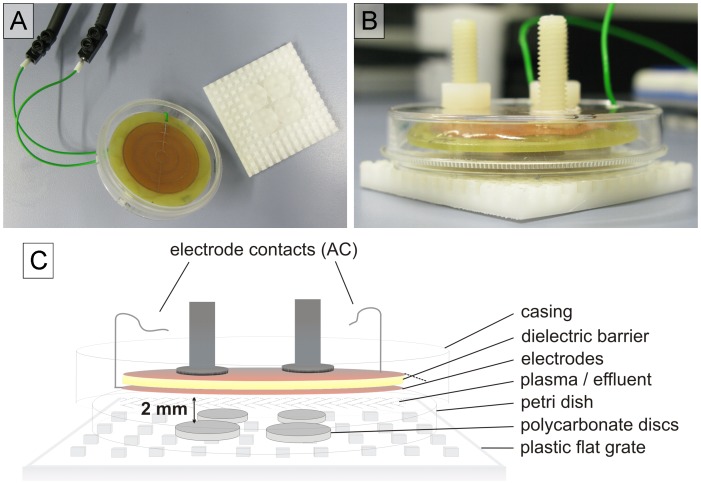
Experimental setup of the SBD-A plasma source. A: Electrode and discs with biofilms on plastic flat grate. B: Configuration of the electrode in action mode. C: Schematic representation of the experimental setup of SBD-A.

**Figure 2:**
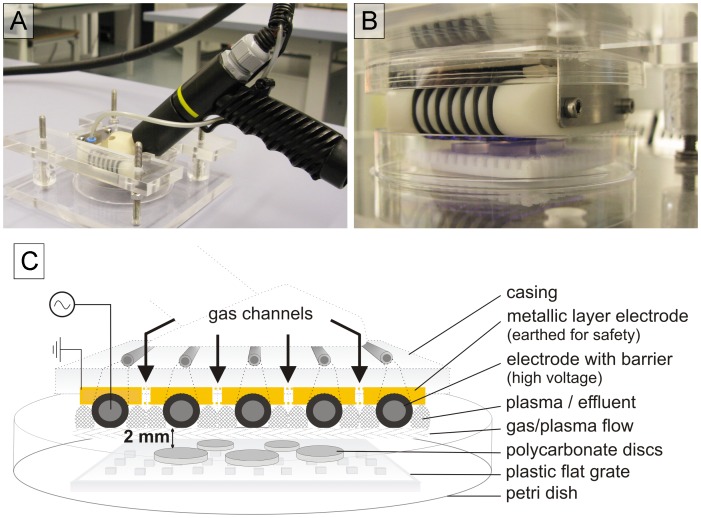
Experimental setup of the SBD-B plasma source. A: Overview of the experimental setup. B: Near focus of the electrode in action mode above the discs with biofilms. C: Schematic representation of the experimental setup of SBD-B in cross section.

Two different types of surface barrier discharges were used (neoplas GmbH, Greifswald, Germany): a structured electrode planar SBD (SBD-A) and a wire electrode SBD (SBD-B). They were both developed and described by Leibniz Institute for Plasma Science and Technology (INP, Greifswald, Germany) [3]. The specifications and physical parameters of both plasma sources are shown in table 1. The dissipated electrical power in both electrode arrangements was measured via the Lissajous method [32]. A Lissajous figure is the *x-y* plot of the charge dissipated into the plasma and the applied voltage. If the plasma is switched on, this plot will yield a parallelogram, whose area resembles the electrical energy E_el_ dissipated into the plasma per duty cycle of the applied voltage. The output power is given by the product of frequency f and dissipated electrical energy. SBD-A is made of a 1.5 mm thick printed circuit board (epoxy glass fiber bulk material), which acts as dielectric barrier, having etched copper electrode structures on its surface (Figure 1). SBD-B has a grounded metallic layer electrode and a high voltage electrode consisting of seven wires of 0.6 mm thickness. Here, silicone insulations around each wire act as dielectric barrier (Figure 2). Both SBD sources are operated with ambient air.

**Table 1:** Operational parameters of SBD-A and SBD-B.

		SBD-A	SBD-B
properties			
**U_SS_ (kV)**		13	8
**applied frequency (kHz)**		20	30
**plasma mode**		burst (2 pulse/ms)	burst (30 pulse/ms)
		= 800 pulse in 400 ms	= 7500 pulse in 250 ms
**working mode**		400 ms on, 1200 ms off	250 ms on, 750 ms off
**dissipated electrical energy (mJ/puls)**		0.6	0.44
**mean power/area (mW/cm^2^)**		0.03	0.18
**mean power/area (mJ/cm^2^ per minute)**		1.8	10.88
**treatment area (cm^2^)**		10	18
**electrode form**		circular	rectangular
**gas flow in slm**		0	0.5

Additionally, SBD-B had applied a flow of compressed air of 0.5 standard litres per minute (slm) through perforations in the grounded electrodes along the border of the insulated powered wire electrodes. The gas flow was controlled by a mass flow controller (MKS Instruments, Germany). The gas temperature of both discharges is close to room temperature at the surface of the substrate [3].

### Measurement of UV Radiation and Ozone

The UV irradiance was measured within a range of 200-400 nm in µW/cm^2^ by optical emission spectroscopy using a fiber optics coupled spectrometer (Avantes AvaSpec-3648, Apeldoorn, Netherlands) at the same distance as applied for antimicrobial plasma treatment (2 mm). The UV exposition in mJ/cm^2^ was determined in accordance with ICNIRP Guidelines [33]. The representation of the graph of UV irradiation and the area below the curve was calculated by using Origin 7.0 (OriginLab Corporation, Northampton, USA). The VUV radiation emission was investigated by means of an apparatus used to determine the VUV radiance [34].

The ozone concentration of both plasma sources for the used treatment conditions was determined by the ozone analyzer (APOA-360, HORIBA, Germany). The detection limit of the ozone analyzer is 16 ppm. Here, the analyzed gas was diluted with a controlled argon gas flow (200 sccm or 400 sccm) to calculate the maximal generated ozone density, which exceeded 16 ppm.

### Cultivation and Evaluation of Biofilms

The Gram-negative strain *Pseudomonas aeruginosa* SG81 (*P. aeruginosa*) and the Gram-positive *Staphylococcus epidermidis* RP62A (*S. epidermidis*) were used. Bacterial cultivation and preparation were performed as described elsewhere [14], [35]. For biofilm cultivation 10% fetal bovine serum (Gibco, Life Technologies) was added to the MEM cell culture medium (Minimum Essential Medium Eagle with L-glutamine, PAA Laboratories, Germany), corresponding to the composition of an artificial wound fluid [6] [36], was used and inoculated with the test bacteria at a final concentration of 10^7^ colony forming units (CFU)/ml. The biofilms were grown on sterile polycarbonate discs (∅ 13 mm, height 3 mm, Arthur Krueger KG, Barsbüttel, Germany), positioned into wells of microplates (Sarstedt AG & Co., Nümbrecht, Germany) and were covered with 800 µl of batch medium. The medium was replaced after 4 h and 24 h. After 24 h discs were turned around to assure similar conditions for both sides of the discs. The biofilms were grown for 48 h at 37°C aerobically and washed once with phosphate buffered saline solution (PBS) to remove unattached bacteria before antiseptic treatments were performed. After treatment, each disc was transferred into sterile wells of microplates and filled with 1 ml PBS. The biofilms were dispensed for 20 min by ultrasound (130 W, Branson 2510 Ultrasonic Cleaner, EMERSON Technologies GmbH & Co. OHG, Dietzenbach, Germany). The antimicrobial effect was determined as difference in the number of the colony forming units (CFU) by microbial culture, as described earlier [14].

### Plasma Treatment

Plasma sources and experimental setup are shown in figures 1 and 2. The prepared biofilm-covered discs were transferred onto sterile plastic flat grates. The plasma sources were positioned 2 mm above the discs. The plasma exposure times were 30, 60, 120, 300 or 600 s, each, on the upper and bottom side of the discs (for this, the discs were turned).

### Treatment with Chlorhexidine Digluconate (CHX)

The prepared biofilm-covered discs were positioned in sterile microplate wells filled with 0.8 ml of 0.1% CHX solution (20% in H_2_O, Fagron GmbH & Co KG, Barsbüttel, Germany) and incubated for 600 s at room temperature. Antiseptic activity was stopped by replacing CHX with 1 ml of inactivation solution (40 g/l Tween 80, 30 g/l saponine, 4 g/l lecitin, 10 g/l sodium dodecyl sulphate, 1 g/l sodium thioglycolate [Serva, Heidelberg, Germany]) and left for 600 s. The efficacy of the inactivation solution was verified according to DIN 1040 [37].

For the cytotoxicity test the 0.1% CHX solution was prepared in culture medium (identical with biofilm growth medium) medium.

### Statistical Analysis

Counted CFU were transformed to log_10_ (CFU/cm^2^). The colony reduction factor (CRF) is defined by the formula:
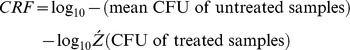



Standard deviations (SD), confidence intervals (CI) and p values were calculated based on log_10_ (CFU/cm^2^) values. Statistical differences between the different treatment times, applied plasma sources and test organisms were analyzed with the Kruskal-Wallis test, followed by a Mann–Whitney *U*-test using statistical analyses system SAS® Enterprise Guide® 4.1 (SAS Institute GmbH, Heidelberg, Germany), where applicable. For the analyses of the cytotoxicity additionally the Bonferroni correction was used.

### Scanning Electron Microscopic Images

For the scanning electron microscopy, the biofilms of *S. epidermidis* and *P. aeruginosa* were prepared in different ways. Discs with *S. epidermidis* biofilms were fixed in 2.5% glutaraldehyde (Sigma-Aldrich, Munich, Germany), solved in 5 mM HEPES (pH 7.4), for 1 h at room temperature and subsequently at 4°C before treatment with amino acid-sucrose-solution (2% arginine, 2% glycine, 2% glutamate, 2% sucrose) followed by guanidine-tannin-solution (2% guanidine, 2% tannin; Sigma-Aldrich, Munich, Germany) for the first 5 min in a microwave and then extended to 1.5 h at room temperature for each solution, 1% osmium tetroxide for 2 h, and 2% uranyl acetate (Plano GmbH, Wetzlar, Germany) for 1.5 h with washing steps in between. After that, samples were dehydrated in a graded series of aqueous ethanol solutions (10–100%) and then critical point-dried via amylacetate and CO_2_.

Discs with *P. aeruginosa* biofilms were prepared as follows. After a fixation step (1 h in 1% glutaraldehyde, 4% paraformaldehyde (Science Services GmbH, Munich, Germany), 100 mM cacodylate buffer [pH 7.4], 1 mM CaCl_2_, 1 mM MgCl_2_, and 50 mM NaN_3_ at room temperature, then 4°C over night), the samples were treated with 2% tannic acid for 1 h, 1% osmium tetroxide for 2 h and after a washing step with 1% osmium tetroxide overnight, and with 2% uranyl acetate for 2 h with washing steps in between. The samples were dehydrated in a graded series of aqueous ethanol solution (10–100%) and then critical point-dried via amylacetate and CO_2_.

Finally, samples of both microorganisms were mounted on aluminium stubs, sputtered with gold/palladium and examined in a scanning electron microscope EVO LS10 SEM (Zeiss, Oberkochen, Germany).

### Cytotoxicity Determination of Plasma

Mouse fibroblasts of the cell line L929 (NCTC clone 929, ATCC CCL-1, USA) were cultivated at 37°C and 5% CO_2_ on round cover glasses (∅ 15 mm, Menzel GmbH, Braunschweig, Germany) in wells of microplates by using 1.5 ml culture medium (identical with biofilm growth medium) per well. The colorimetric MTT (3-(4,5-Dimethylthiazol-2-yl)-2,5-diphenyltetrazolium bromide; Sigma-Aldrich, Munich, Germany) assay in accordance with DIN EN ISO 10993-5 [38] was used to determine the cytotoxic effects of CHX and the generated plasma (parameters as used for biofilm treatment, see above) respectively. The MTT assay was based on the selective ability of living cells to reduce the salt MTT to formazan by dehydrogenases [39].

For plasma treatment, discs with cells were positioned on plastic flat grates in a petri dish at a distance of 2 mm to the electrodes of the plasma sources. The edges of the discs were surrounded with medium to have wet conditions to avoid drying. For CHX treatment, discs were transferred into a separate 24-well-microplate and the cells were covered with 300 µl of 0.1% CHX solution for 30, 60, 150, 300 and 600 s, respectively. Then, the discs were washed two times with fresh cell culture media.

The cytotoxicity test was carried out after 48 h of cell cultivation (4 samples with plasma and 3 samples with CHX, respectively). After plasma treatment, 5 µg/ml gentamicin and 0.25 µg/ml amphotericin B were added to the medium to inactivate possible bacterial contamination during treatment. Three samples were used as untreated control, on open air beside the plasma treatment and 6 samples incubated with fresh media only for the CHX treatment.

For the MTT assay the discs were transferred into a new microplate. The wells were filled with 0.5 ml MTT media (500 µg MTT/ml culture media) and incubated at 37°C with 5% CO_2_ for 3 h. Next, MTT media was replaced by 0.04 M HCl in 2-propanol (97%, Merck KGaA, Darmstadt, Germany) for 1 h at room temperature on an agitator at 300 rpm (Polymax, Heidolph, Germany) to elute the formazan crystals. 200 µl of the eluate of each well were transferred into wells of a 96-well microplate and measured by spectrometry at 540 nm (PowerWave HT, BioTek Instruments GmbH, Bad Friedrichshall, Germany). Besides the untreated control, a positive control with Triton™ X-100 (10% in H_2_O, Sigma-Aldrich, Munich, Germany) was used to verify the test.

## Results

Antimicrobial effects were shown for all treatment variations (Tables 2 and 3) and were statistically significant compared to the control. The efficacy depended on treatment time and differed between SBD-A and SBD-B. Generally, SBD-A showed a higher CRF than SBD-B and the CRF increased with increasing treatment time. The difference between both plasma sources is statistically significant for every treatment applied to *P. aeruginosa*, irrespective of the duration, and within a treatment time of 150 and 600 s for *S. epidermidis*. The range of CRF of SBD-A electrode was 1.4 log_10_ (CFU/cm^2^) up to 7.1 log_10_ (CFU/cm^2^), and of SBD-B electrode 1.1 log_10_ (CFU/cm^2^) up to 3.8 log_10_ (CFU/cm^2^) for *P. aeruginosa*, and 0.7 log_10_ (CFU/cm^2^) to 3.4 log_10_ (CFU/cm^2^) as well as 0.6 log_10_ (CFU/cm^2^) to 2.7 log_10_ (CFU/cm^2^) for *S. epidermidis*.

**Table 2:** Antiseptic treatment of *Pseudomonas aeruginosa* SG81 biofilms.

		SBD-A				SBD-B			
			95% CI limits				95% CI limits		
treatment	n	CRF ± SD	lower	upper	n	CRF ± SD	lower	upper	p
**plasma - 30 s**	20	**1.44**±0.41^c^ *	1.25	1.63	26	**1.16**±0.60^c^ *	0.92	1.40	0.0037
**plasma - 60 s**	24	**1.78**±0.70	1.48	2.07	20	**1.32**±0.41^c^	1.13	1.51	0.0267
**plasma - 150 s**	22	**2.60**±0.77^c^	2.26	2.94	20	**2.06**±0.66*	1.76	2.37	0.0136
**plasma - 300 s**	23	**4.83**±2.19^c^ *	3.88	5.77	20	**2.98**±0.96^c^ *	2.53	3.43	0.0050
**plasma - 600 s**	16	**7.11**±1.17^c^ *	6.48	7.73	12	**3.81**±1.51^c^	2.85	4.77	0.0001
p		0.0001				0.0001			0.0001
**CHX 0.1%**	35	**1.72**±0.46	1.56	1.88					
**control**	38	**0.00**±0.15	−0.05	0.05					
p		0.0001				0.0001			0.0001

The analytical results by the Number of samples (n), Colony reduction factor (CRF) in log_10_ (CFU/cm^2^) ± Standard Deviation (SD), lower and upper 95% confidence limits (CI) after exposure to air plasma for 30–600 s treatment time respectively and 0.1% CHX after 600 s exposure time and untreated control of *Pseudomonas aeruginosa* SG81 biofilms [p-values of omnibus tests (Kruskal-Wallis) and two-sample tests (Whitney *U*); statistical significance: α = 0.05].

c significantly different from CHX.

* significantly different from the respective treatment time of *Staphylococcus epidermidis* RP62A.

**Table 3:** Antiseptic treatment of *Staphylococcus epidermidis* RP62A biofilms.

		SBD-A				SBD-B			
			95% CI limits				95% CI limits		
treatment	n	CRF ± SD	lower	upper	n	CRF ± SD	lower	upper	p
**plasma - 30 s**	20	**0.66**±0.64^c^ *	0.36	0.97	12	**0.57**±0.45^c^ *	0.28	0.85	0.8763
**plasma - 60 s**	22	**1.55**±1.04	1.09	2.01	20	**1.23**±0.40	1.05	1.42	0.5795
**plasma - 150 s**	22	**2.32**±1.19^c^	1.79	2.85	20	**1.45**±0.56^c^ *	1.19	1.71	0.0219
**plasma - 300 s**	22	**2.77**±1.27^c^ *	2.20	3.33	20	**2.04**±0.58^c^ *	1.77	2.31	0.0698
**plasma - 600 s**	16	**3.38**±0.87^c^ *	2.92	3.85	12	**2.69**±0.98^c^	2.07	3.31	0.0459
p		0.0001				0.0001			0.0001
**CHX 0.1%**	32	**1.14**±0.73	0.88	1.41					
**control**	30	**0.00**±0.30	−0.11	0.11					
p		0.0001				0.0001			0.0001

The analytical results by the Number of samples (n), Colony reduction factor (CRF) in log_10_ (CFU/cm^2^) ± Standard Deviation (SD), lower and upper 95% confidence limits (CI) after exposure to air plasma for 30–600 s treatment time and 0.1% CHX after 600 s exposure time and untreated control of *Staphylococcus epidermidis* RP62A biofilms [p-values of omnibus tests (Kruskal-Wallis) and two-sample tests (Whitney *U*); statistical significance: α = 0.05].

c significantly different from CHX.

* significantly different from the respective treatment time of *Pseudomonas aeruginosa* SG81.

**Figure 3:**
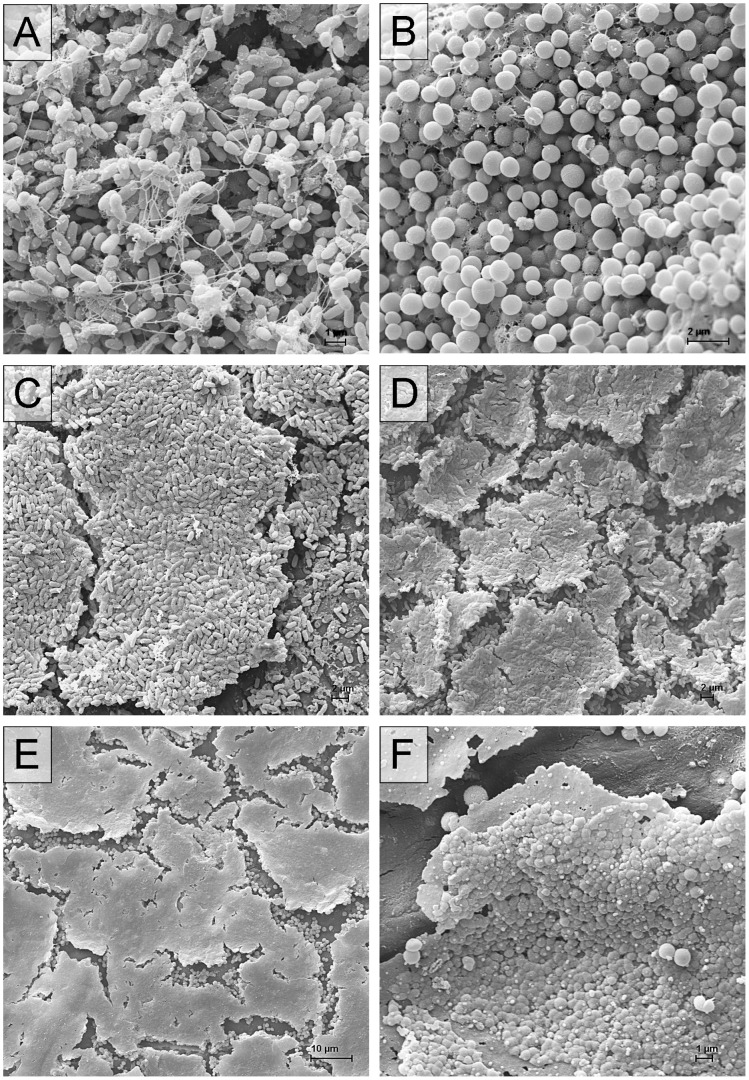
Scanning electron micrographs of untreated and air plasma treated biofilms on polycarbonate discs. A) untreated biofilm of *Pseudomonas aeruginosa* SG81 (5000-fold), B) untreated biofilm of *Staphylococcus epidermidis* RP62A (5000-fold), C) *Pseudomonas aeruginosa* SG81 biofilms after 300 s of air plasma treatment by SBD-A (2000-fold) and D) by SBD-B (1500-fold) as well as E) *Staphylococcus epidermidis* RP62A biofilms after 300 s of air plasma treatment by SBD-A (1000-fold) and F) by SBD-B (5000-fold).

**Figure 4:**
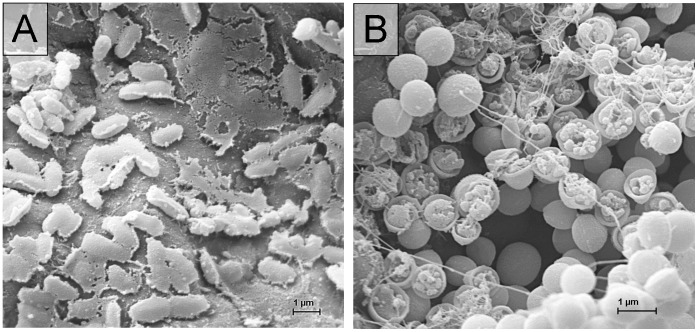
Scanning electron micrographs of cell morphology damaged biofilm bacteria on polycarbonate discs after 300 s of air plasma treatment with A) *Pseudomonas aeruginosa* SG81 by SBD-B (7000-fold) and B) *Staphylococcus epidermidis* RP62A by SBD-A (10000-fold).

The scanning electron micrographs in figure 3 show untreated (Figure 3 A and B) as well as plasma treated biofilms of *P. aeruginosa* (Figure 3 C and D) and *S. epidermidis* (Figure 3 E and F). Untreated biofilms of *P. aeruginosa* show stable rod-shaped and *S. epidermidis* spherical cell morphology (Figure 3 A and B). After plasma treatment the micrographs showed that the biofilms were covered by a flat layer (Figure 3 E) or by conglomerated cells (Figures 3 C, D and F). Figure 4 shows destructed but non-conglomerated cells in different morphology for *P. aeruginosa* and *S. epidermidis*. Cells of *P. aeruginosa* show mushy (Figure 4 A) and *S. epidermidis* show split shape (Figure 4 B).

**Figure 5:**
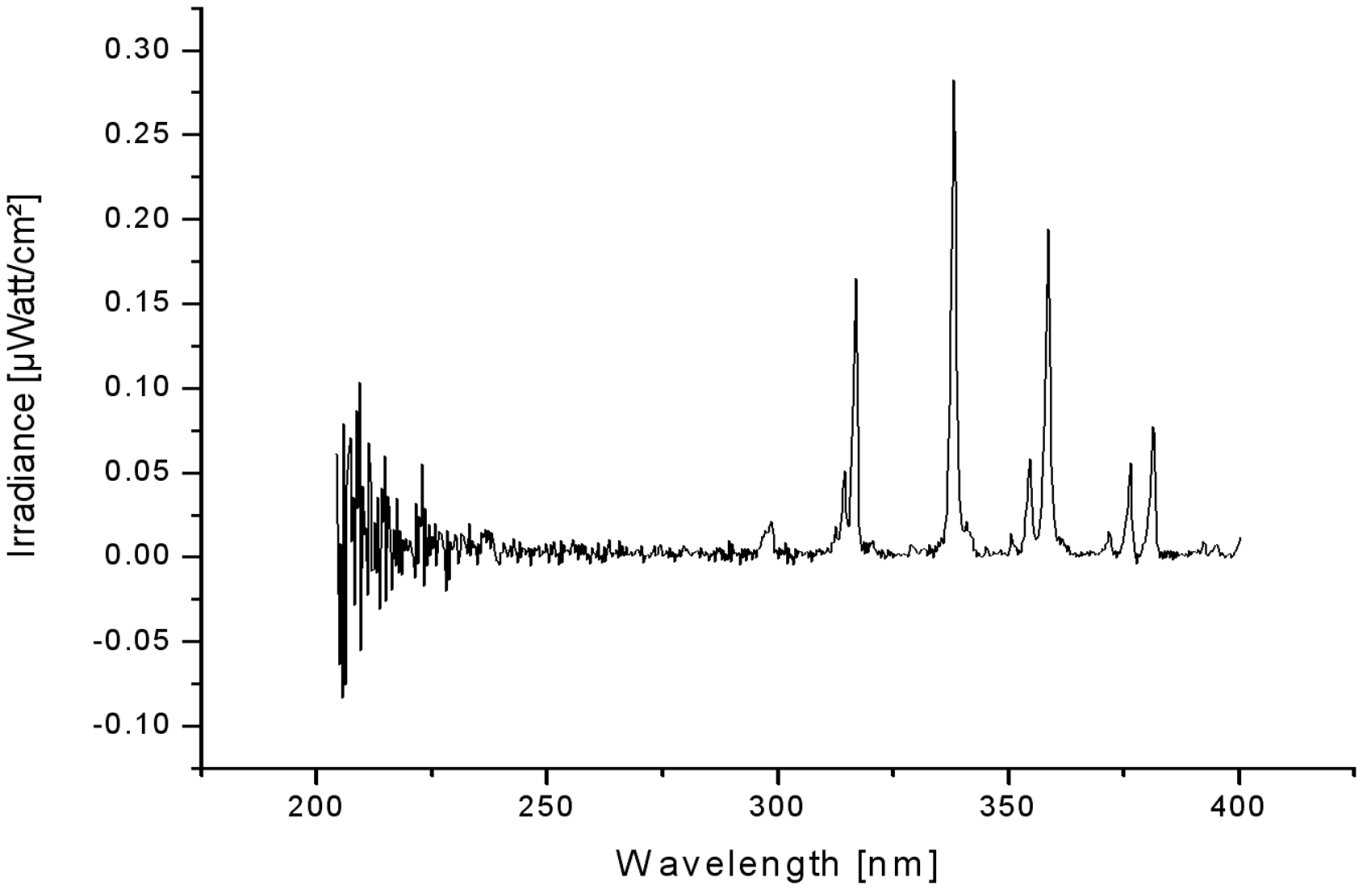
Spectrometric graph of irradiance by SBD-B generated air plasma within 300 s of exposure time between 200 and 400 nm.

The maximum UV irradiation measured between 200–400 nm was 2.5 µW/cm^2^ by SBD-B during 300 s of exposure time. Here, the emission spectrum is shown in Figure 5. The UV radiation of SBD-A and the measured VUV radiation from 115 to 200 nm of both plasma sources is below the detection limit.

The measured ozone density of SBD-A increased to a maximum of 34.7 ppm and SBD-B of 18.8 ppm within 80 s plasma exposure time.

The cytotoxicity of both plasma sources determined by the MTT-assay is similar (Table 4). Compared to the untreated control, the viability exceeds in this case 50% after plasma treatment times from 30 s to 150 s, whereas it decreases below 50% after 300 s and 600 s treatment time. The gas flow of SBD-B showed no devitalising effects. Compared to the control values, the viability after the cytotoxicity test of 0.1% CHX was less than 50% at treatment times between 30 to 600 s (Table 5). The differences between plasma of SBD A for 600 s treatment time and CHX treated cells for 30 to 600 s treatment time to the untreated control as well as the differences between the plasma treated cells to the CHX treated cells for both plasma sources at any treatment time were statistically significant.

**Table 4:** Cytotoxicity of air plasma by SBD-A and SBD-B on L929 cell line (mouse fibroblasts).

		SBD-A					SBD-B			
treatment	n	mean ± SD			ratio to control (%)	n	mean ± SD			ratio to control (%)
**plasma - 30 s**	3	0.090 ± 0.022			**89.0**	2	0.094 ± 0.004			**92.6**
**plasma - 60 s**	4	0.096 ± 0.005			**94.1**	4	0.075 ± 0.004			**73.9**
**plasma - 150 s**	4	0.075 ± 0.022			**74.1**	4	0.085 ± 0.014			**83.3**
**plasma - 300 s**	4	0.036 ± 0.024			**35.0**	4	0.044 ± 0.032			**43.3**
**plasma - 600 s**	4	0.004 ± 0.003			**4.2**	4	0.043 ± 0.028			**42.6**
**gas flow - 300 s**						2	0.088 ± 0.010			**86.7**
**control**	4	0.102 ± 0.011								

Measured values of MTT-Assay after 30–600 s treatment time of air plasma by SBD-A and SBD-B with the Number of samples (n), mean, standard deviation (SD), and the cell viability as ratio in comparison to the control in percent.

**Table 5:** Cytotoxicity of 0.1% chlorhexidine digluconate solution on L929 cell line (mouse fibroblasts).

CHX (0.1%)					
treatment	n	mean ± SD			ratio to control (%)
**CHX - 30 s**	3	0.046 ± 0.029			**17.3**
**CHX - 60 s**	3	0.021 ± 0.017			**7.7**
**CHX - 150 s**	3	0.069 ± 0.013			**25.9**
**CHX - 300 s**	3	0.016 ± 0.011			**6.0**
**CHX - 600 s**	3	0.007 ± 0.002			**2.5**
**control**	6	0.267 ± 0.076			

Measured values of MTT-Assay after 30–600 s treatment time with 0.1% of chlorhexidine dicluconate solution (in culture media) with the Number of samples (n), mean, standard deviation (SD), and the cell viability as ratio in comparison to the control in percent.

## Discussion

Currently, no standard model for testing of antiseptics against microorganisms in biofilms on various substrates is defined in literature. The used biofilm model with incubation times of 48 h at 37°C and early replacement of medium showed optimal results for stable and standardized biofilms in previous experiments. The used test microorganisms *Pseudomonas aeruginosa* SG81 and *Staphylococcus epidermidis* RP62A are often used for biofilm analyses and tests [40], [41], [42], [43], [44]. They are relevant biofilm forming pathogens in chronic wounds or device related infections [29], [45]. Furthermore, both organisms have been partly isolated from clinical cases as drug-resistant strains [46], [47]. The number of infections with drug-resistant microorganisms is increasing worldwide [48] which underlines the necessity to develop new eradication strategies.

The medium contained 10% fetal bovine serum (corresponding to the composition of an artificial wound fluid [6] [36]) and was used as growth medium to investigate biofilms under more realistic wound-like conditions. For the experiments, polycarbonate discs as substrate were used because polycarbonate is widely used in medicine [49] and is also suited to culture biofilms [50]. The biofilms of the untreated controls had to result in more than 6 log_10_ (CFU/cm^2^) after dispersion by ultrasound to ensure a sufficient biofilm growth. The untreated controls in the study showed mean values at 7.89±0.38 log_10_ (CFU/cm^2^) for *P. aeruginosa* and 6.85±0.41 log_10_ (CFU/cm^2^) for *S. epidermidis* and were used to calculate the CRFs after antiseptic treatment. The antiseptic CHX was used as positive control and as control of the homogeneity of the biofilm sensitivity to antiseptic treatment, because 0.1% CHX showed comparable or higher antimicrobial efficacy against *in vitro* biofilms than the standard wound antiseptic polihexanide [14] and CHX is also used as antiseptic in wound dressing [31]. Furthermore, 0.1% CHX is also recommended for biofilm inhibition in the mouth cavity [51] and was often used as positive control for plasma treatments on biofilms [14], [15], [52].

Biofilm covered discs used for plasma treatment were transferred onto a plastic flat grate in order to keep the contact area minimal, since both sides of the discs were exposed to the plasma for the respective treatment time in order to treat the whole colonised area. A distance of 2 mm between the discs and electrode had been determined as optimal for treatment by the microbial agar test method [35] by pre-tests.

The two investigated SBD plasma sources were selected because large area discharges could be suitable to treat homogenously superficial infected wounds on skin. Additionally, the shape of SBD-B electrode can be customized to different surface structures and application forms for possible plasma treatment on skin or wounds [53]. Furthermore, the temperature of the electrodes during plasma generation was equal to the adjacent room temperature for SBD-A and close to body temperature for SBD-B (fluoroptic temperature measurement), and the maximally generated UV radiation at 2.5 µW/cm^2^ corresponded to an UV-exposition of less than 0.05 mJ/cm^2^ which is extremely low and non-hazardous according to the ICNIRP Guidelines on limits of exposure to ultraviolet radiation [33]. The graphical peaks were NO at 297 nm and excited N_2_ at 314, 316.7, 338, 354.5, 358.5, 376.5 and 381 nm. The irregular peaks between 200 and 225 nm are due to increased noise caused by the detection limit of the spectrometer used. Both plasma sources are operated with or in air. Therefore VUV radiation is quickly absorbed in this atmosphere [33] and was too low for detection in the used application setting.

Advantages of air as working gas are that oxygen and nitrogen for ROS and RNS generation are continuously available, no separate gas tanks or gas connections are necessary, thus it is easier applicable and cheaper than commonly used inert gases. The gas flow of SBD-B was applied to enhance the homogeneous distribution of the reactive plasma components.

Both SBDs showed different inactivation efficacies (Tables 2 and 3). SBD-A is more effective than SBD-B for all treatment times on *P. aeruginosa* (p<0.027) and after 600 s on *S. epidermidis* biofilms (p = 0.046). The difference was obvious, in particular for *P. aeruginosa* where the maximum CRF of SBD-A was 7.1 log_10_ (CFU/cm^2^) but only 3.8 log_10_ (CFU/cm^2^) by SBD-B. However, for *S. epidermidis* the maximum CRF of SBD-A was 3.4 log_10_ (CFU/cm^2^) and 2.7 log_10_ (CFU/cm^2^) by SBD-B.

An explanation could be the formation of differently dense cell detritus layers on top of the biofilms which inhibits deeper plasma effects on biofilm. Scanning electron micrographs of *P. aeruginosa* biofilms after 300 s of exposure to plasma (Figure 3 C, D) showed that the plasma treatment seems to produce different dense detritus layers. Maybe, for SBD-A, the reactive plasma-gas compound could work in deeper biofilm regions of *P. aeruginosa* than SBD-B despite of gentle gas flow, because the reactive products which are responsible for the antimicrobial effect in deeper layers could possibly generated at different concentrations. The micrographs additionally showed that the detritus layer on *S. epidermidis* biofilm after plasma treatment with both plasma sources was very dense (Figure 3 E, F). The scanning electron micrographs revealed non-destructed bacteria visible between the clods of the disrupted biofilms. Those bacteria are probably not inactivated by plasma. It can be deduced that the detritus layer on the biofilm surface restricts the plasma efficiency. This could be an explanation for the increased standard deviation after treatment of *P. aeruginosa* biofilms on the one hand and the limited antimicrobial effect against *S. epidermidis* on the other hand.

The detritus layer seems to be the result of coagulated biofilm matrix as well as cytoplasm after bacterial cell wall disruption by reactions with the generated plasma products. Differences in disrupted cell morphology were visible between the Gram-negative *P. aeruginosa* and the Gram-positive *S. epidermidis*, whereas *S. epidermidis* as a Gram-positive strain with a cell wall containing a thick peptidoglycan layer is opened and *P. aeruginosa* as a Gram-negative strain with an outer cell membrane and only a thin peptidoglycan layer loses the three-dimensionality to a flat irregular morphology (Figure 4) after longer treatment time. That corresponds to observations of other authors [54], [55]. Similar cell wall disruption of *S. epidermidis* was shown for *Candida albicans*, a unicellular fungi with a glycan rich cell wall, in a former study [52]. That supports the supposition of a cell wall influenced dependence of the antimicrobial mechanism of plasma [55]. For comparison, figure 3 shows untreated biofilms of *P. aeruginosa* and *S. epidermidis*. The “clods” of disrupted biofilms visible in scanning electron micrographs of figure 3 C–F were probably caused by drying due to the plasma treatment or the drying steps by SEM procedure.

Also, a different structure of biofilm matrix between *S. epidermidis* and *P. aeruginosa* could explain the different results of SBD-B treatment on both test organisms but not the different results between both plasma sources against *P. aeruginosa* biofilms. The higher power per area of SBD-B at 0.18 mW/cm^2^ compared to SBD-A electrode at 0.03 mW/cm^2^ cannot be decisive for antimicrobial results. Here, effluent distribution over the substrate or different plasma-gas compounds by different plasma generation seems to influence the biological effects perceptibly. To clarify the different efficacy, the generated ozone concentration of both devices was determined. The results obtained using the experimental setup showed a higher share of O_3_ generated by SBD-A. Moore et al. [56] showed a higher susceptibility of Gram-negative bacterial strains to O_3_ which could explain the unexpected high antimicrobial effect of SBD-A on biofilms of *P. aeruginosa*. Additionally, a study of Zhang et al. [57] suggests that O_3_ play a minor role for inactivation of the Gram-positive *Staphylococcus aureus*. However, other reactive species in the gas or different secondary products on the substrate cannot be excluded to be decisive for the observed effect.

The efficacy of both plasma devices is comparable to the results of other studies. An argon plasma jet (kinpen 09), for instance, induced a reduction of 5.4 log_10_ for *P. aeruginosa* and 3 log_10_ for *S. epidermidis* after 300 s exposure time [58]. A helium plasma jet caused a reduction of 4 log_10_ of *P. aeruginosa* after 240 s exposure time [59]. Otherwise, only approximately 1 log_10_ reduction were reached by 600 s blowing of a DBD generated air plasma (in a separate box) on biofilm embedded Staphylococci [60] or 2 log_10_ reduction against *S. epidermidis* after 600 s by a sprayed gliding discharge plasma of humidified air [61]. Here, the distance between the electrodes and the target was extended. Those differences of biological effectiveness underline the important influence of the distance between the plasma generating electrodes and bacteria.

Generally, in biofilms *P. aeruginosa* SG81 was more sensitive than *S. epidermidis* RP62A to air plasma in that study, which could be due to the oxidation of membrane lipids by ROS of the Gram-negative *P. aeruginosa* strain [62], [63]. However, a previous study showed that other conditions or plasma setups can result in different effects between Gram-positive and Gram-negative bacteria spread on agar plates by treatment with air plasma [64]. In comparison to the untreated control all antiseptic treatments exhibited a superior efficacy towards the used strains (p<0.001) which is statistically significant. In comparison to 600 s of CHX treatment, the CRFs of plasma had shown a statistically higher value already after 150 s of treatment for both strains (all p<0.028), except for SBD-B at 150 s treatment time for *P. aeruginosa* (p = 0.057).

Additional, results of the cytotoxicity test by MTT assay showed that the average viability of the cells did not decrease below 50% until 150 s of exposure to plasma for both plasma sources. The 50% limit was chosen based on the XTT assay, which is similar to the MTT assay, described in ISO 10993-5. Here, a loss of the cell viability between 70% to 50% compared to the control is described as moderate cytotoxicity [38]. That suggests an acceptable tissue tolerability within individual limited treatment times. Here, the antimicrobial efficacy on biofilms was approximately 1.5 log_10_ (CFU/cm^2^) for *S. epidermidis* and 1.8 to 2 log_10_ (CFU/cm^2^) for *P. aeruginosa*. That is low but reaches the antimicrobial effect of CHX on biofilms. Additionally, the cell viability of 0.1% CHX was clearly less than 50% compared to the control for 30 s to 600 s treatment time while the antimicrobial efficacy with a CRF at 1.1 log_10_ (CFU/cm^2^) for *S. epidermidis* and 1.7 log_10_ (CFU/cm^2^) for *P. aeruginosa* is lower or comparable to the air plasma treatment times for cytotoxicity acceptable doses.

Beside the antimicrobial effect on biofilms, the stimulation of wound healing processes is necessary to heal chronic wounds. An enhanced cell growth by air plasma was reported by other authors [22], [65]. Plasma supported wound healing was also demonstrated in clinical or preclinical practice [66], [67], [68]. These facts could promote a therapeutic application form of the investigated plasma sources as support for wound healing processes with slight antimicrobial effect (in tissue tolerable doses).

For future therapy options, it is indispensable to consider the limitations of the presented plasma application, i.e., side effects like the observed cell detritus. Also, the treatment time must be correctly chosen as plasma treatment could turn toxic if applied too long.

The present study recommends short treatment times (<60 s) on biofilms to reduce possible dense cell detritus layers which complicate a whole biofilm inactivation and to minimize the toxic risks. Maybe combination of plasma with antiseptic solutions could be a treatment option. Promising results for combinations of chemical antiseptics with plasma were presented [19].

The study has some limitations. We used 48-h-old monospecies *in vitro* biofilms grown under controlled conditions to investigate the antimicrobial efficacy of plasma, however natural and wound multi-species biofilms *in vivo* are more complex than our monospecies biofilms [21]. The plasma treatment is to be realised under defined exposure distances from the specimen for standardization, while biological surfaces to be treated often have variable structures. In order to allow the comparability, the cytotoxicity test was carried out with a standard murine fibroblast cell line for *in vitro* cytotoxicity tests [38] and not with human epithelial cells. Consequently, these results are only for orientation. Additional, plasma effects on real wounds are influenced by individual wound secret and local immunologic cells, so more complex reactions and influences than under laboratory conditions are expected.

### Conclusion

A high antimicrobial effect on biofilms with *P. aeruginosa* and *S. epidermidis* is confirmed for both plasma sources used. Here, SBD-A is more effective than SBD-B. Taking into consideration the cytotoxicity, it is recommended to limit the application time to 60 s for SBD-A and 150 s for SBD-B. Within these exposure times both plasma sources meet important preconditions of tissue tolerability such as temperature, UV exposure and cytotoxicity. Their antimicrobial efficacy is comparable to the efficacy of 0.1% chlorhexidine digluconate solution, the antiseptic gold standard for dental biofilm treatment, however with significantly lower cytotoxicity of the used plasma. In addition, if these plasma sources prove suitable to expedite wound healing processes, they are likely to be successfully applied in wound care management, too.

Formation of bacterial cell detritus on biofilm surface can limit the antimicrobial effect which depends on plasma source, plasma exposition time and treated type of microorganism. Here, the Gram-negative bacterium was more sensitive to air plasma than the Gram-positive strain. Future investigations into the antimicrobial mechanisms of plasma could elucidate the differences of both generated “types” of air plasma by SBD-A and -B.

## References

[1] Daeschlein G, Woedtke Tv, Kindel E, Brandenburg R, Weltmann K-D, et al (2010) Antibacterial Activity of an Atmospheric Pressure Plasma Jet Against Relevant Wound Pathogens in vitro on a Simulated Wound Environment. Plasma Process Polym 7: 224–230.

[2] Kramer A, Lindequist U, Weltmann KD, Wilke C, von Woedtke T (2008) Plasma Medicine - its perspective for wound therapy. GMS Krankenhhyg Interdiszip 3 Doc16. 20204118 PMC2831519

[3] Weltmann KD, Kindel E, von Woedtke T, Hähnel M, Stieber M, et al (2010) Atmospheric-pressure plasma sources: Prospective tools for plasma medicine. Pure Appl Chem 82: 1223–1237.

[4] Burmølle M, Thomsen TR, Fazli M, Dige I, Christensen L, et al (2010) Biofilms in chronic infections - a matter of opportunity - monospecies biofilms in multispecies infections. FEMS Immunol Med Microbiol 59: 324–336. 20602635 10.1111/j.1574-695X.2010.00714.x

[5] Lademann O, Kramer A, Richter H, Patzelt A, Meinke MC, et al (2011) Skin Disinfection by Plasma-Tissue Interaction: Comparison of the Effectivity of Tissue-Tolerable Plasma and a Standard Antiseptic. Skin Pharmacol Physiol 24: 284–288. 21709431 10.1159/000329913

[6] Müller G, Kramer A (2008) Biocompatibility index of antiseptic agents by parallel assessment of antimicrobial activity and cellular cytotoxicity. J Antimicrob Chemother 61: 1281–1287. 18364400 10.1093/jac/dkn125

[7] Hammann A, Hübner N-O, Bender C, Ekkernkamp A, Hartmann B, et al (2010) Antiseptic Efficacy and Tolerance of Tissue-Tolerable Plasma Compared with Two Wound Antiseptics on Artificially Bacterially Contaminated Eyes from Commercially Slaughtered Pigs. Skin Pharmacol Physiol 23: 328–332. 20588084 10.1159/000314724

[8] Lademann J, Richter H, Schanzer S, Patzelt A, Thiede G, et al (2012) Comparison of the antiseptic efficacy of tissue-tolerable plasma and an octenidine hydrochloride-based wound antiseptic on human skin. Skin Pharmacol Physiol 25: 100–106. 22301799 10.1159/000335558

[9] Vinh DC, Embil JM (2005) Device-related infections: a review. J Long Term Eff Med Implants 15: 467–488. 16218897 10.1615/jlongtermeffmedimplants.v15.i5.20

[10] Bjarnsholt T, Kirketerp-Møller K, Jensen PØ, Madsen KG, Phipps R, et al (2008) Why chronic wounds will not heal: a novel hypothesis. Wound Repair Regen 16: 2–10. 18211573 10.1111/j.1524-475X.2007.00283.x

[11] Kramer A, Hübner N-O, Assadian O, Below H, Bender C, et al (2009) Chances and perspectives of the plasma medicine by use of Tissue Tolerable Plasma (TTP). GMS Krankenhhyg Interdiszip 4 Doc10.

[12] Lademann J, Richter H, Alborova A, Humme D, Patzelt A, et al (2009) Risk assessment of the application of a plasma jet in dermatology. J Biomed Opt 14: 054025. 19895127 10.1117/1.3247156

[13] Weltmann KD, Kindel E, Brandenburg R, Meyer C, Bussiahn R, et al (2009) Atmospheric Pressure Plasma Jet for Medical Therapy: Plasma Parameters and Risk Estimation. Contrib Plasma Phys 49: 631–640.

[14] Hübner NO, Matthes R, Koban I, Rändler C, Müller G, et al (2010) Efficacy of chlorhexidine, polihexanide and tissue-tolerable plasma against *Pseudomonas aeruginosa* biofilms grown on polystyrene and silicone materials. Skin Pharmacol Physiol 23: 28–34. 20829659 10.1159/000318265

[15] Koban I, Holtfreter B, Hübner N-O, Matthes R, Sietmann R, et al (2011) Antimicrobial efficacy of non-thermal plasma in comparison to chlorhexidine against dental biofilms on titanium discs in vitro - proof of principle experiment. J Clin Periodontol 38: 956–965. 21762196 10.1111/j.1600-051X.2011.01740.x

[16] Hübner N, Matthes R, Gruman D, Holtfreter S, Bröker B, et al. Antimicrobial efficacy of low-temperature plasma against 65 genetically characterized *Staphylococcus aureus* isolates including PVL-positive, MRSA and c-MRSA strains; 2010 3–5 November; Valladolid (Spain).

[17] Bender C, Partecke LI, Kindel E, Döring F, Lademann J, et al (2011) The modified HET-CAM as a model for the assessment of the inflammatory response to tissue tolerable plasma. Toxicol In Vitro 25: 530–537. 21111803 10.1016/j.tiv.2010.11.012

[18] Lademann J, Ulrich C, Kluschke F, Patzelt A, Czaika VA, et al. Effects of tissue-tolerable plasma on chronic wound treatment compared to a modern conventional liquid antiseptic.; 2012 28–30 November; Copenhagen, Denmark. ( (2013) Abstract. Skin Research and Technology 19: e552–e592.

[19] Bender C, Hübner NO, Weltmann KD, Scharf C, Kramer A (2012) Tissue Tolerable Plasma and Polihexanide: Are Synergistic Effects Possible to Promote Healing of Chronic wounds? In Vivo and In Vitro Results. In: Machala Z, Hensel K, Akishev Y, editors. NATO Science for Peace and Security Series A: Chemistry and Biology. Dordrecht: Springer Netherlands. 479p.

[20] Park GY, Park SJ, Choi MY, Koo IG, Byun JH, et al (2012) Atmospheric-pressure plasma sources for biomedical applications. Plasma Sources Sci Technol 21: 043001.

[21] James GA, Swogger E, Wolcott R, Pulcini E, Secor P, et al (2008) Biofilms in chronic wounds. Wound Repair Regen 16: 37–44. 18086294 10.1111/j.1524-475X.2007.00321.x

[22] Kalghatgi S, Kelly CM, Cerchar E, Torabi B, Alekseev O, et al (2011) Effects of Non-Thermal Plasma on Mammalian Cells. PLoS One 6: e16270. 21283714 10.1371/journal.pone.0016270 PMC3025030

[23] Reuter S, Tresp H, Wende K, Hammer MU, Winter J, et al (2012) From RONS to ROS: Tailoring Plasma Jet Treatment of Skin Cells. IEEE Trans Plasma Sci 40: 2986–2993.

[24] Nosenko T, Shimizu T, Morfill GE (2009) Designing plasmas for chronic wound disinfection. New J Phys 11: 115013.

[25] Oehmigen K, Hähnel M, Brandenburg R, Wilke C, Weltmann KD, et al (2010) The Role of Acidification for Antimicrobial Activity of Atmospheric Pressure Plasma in Liquids. Plasma Process Polym 7: 250–257.

[26] Joshi SG, Cooper M, Yost A, Paff M, Ercan UK, et al (2011) Nonthermal Dielectric-Barrier Discharge Plasma-Induced Inactivation Involves Oxidative DNA Damage and Membrane Lipid Peroxidation in Escherichia coli. Antimicrob Agents Chemother 55: 1053–1062. 21199923 10.1128/AAC.01002-10 PMC3067084

[27] Laroussi M, Leipold F (2004) Evaluation of the roles of reactive species, heat, and UV radiation in the inactivation of bacterial cells by air plasmas at atmospheric pressure. Int J Mass Spectrom 233: 81–86.

[28] Fazli M, Bjarnsholt T, Kirketerp-Møller K, Jørgensen B, Andersen AS, et al (2009) Nonrandom Distribution of *Pseudomonas aeruginosa* and *Staphylococcus aureus* in Chronic Wounds. J Clin Microbiol 47: 4084–4089. 19812273 10.1128/JCM.01395-09 PMC2786634

[29] Uckay I, Pittet D, Vaudaux P, Sax H, Lew D, et al (2009) Foreign body infections due to *Staphylococcus epidermidis* . Ann Med 41: 109–119. 18720093 10.1080/07853890802337045

[30] Moshrefi A (2002) Chlorhexidine. J West Soc Periodontol Periodontal Abstr 50: 5–9. 12049062

[31] Muangman P, Nitimonton S, Aramwit P (2011) Comparative Clinical Study of Bactigras and Telfa AMD for Skin Graft Donor-Site Dressing. Int J Mol Sci 12: 5031–5038. 21954342 10.3390/ijms12085031 PMC3179149

[32] Wagner HE, Brandenburg R, Kozlov KV, Sonnenfeld A, Michel P, et al (2003) The barrier discharge: basic properties and applications to surface treatment. Vacuum 71: 417–436.

[33] ICNIRP (2004) Guidelines on limits of exposure to ultraviolet radiation of wavelengths between 180 nm and 400 nm (incoherent optical radiation). Health Phys 87: 117–186. 10.1097/00004032-200408000-00006 15257218

[34] Lange H, Foest R, Schafer J, Weltmann KD (2009) Vacuum UV Radiation of a Plasma Jet Operated With Rare Gases at Atmospheric Pressure. IEEE Trans Plasma Sci 37: 859–865.

[35] Matthes R, Hübner N, Bender C, Koban I, Weltmann K, et al (2010) Screening test for quality control of surface barrier discharged plasma sources with the microorganism-agar test (MAT). GMS Krankenhhyg Interdiszip 5: DOC02. 20941344 10.3205/dgkh000145 PMC2951102

[36] Campbell KE, Keast D, Woodbury G, Houghton P (2003) Wear time in two hydrocolloid dressings using a novel in-vivo model. Wounds 15: 40–48.

[37] DIN EN 1040 D (2006) DIN EN 1040:2006-03 Chemical disinfectants and antiseptics - Quantitative suspension test for the evaluation of basic bactericidal activity of chemical disinfectants and antiseptics - Test method and requirements (phase 1); German version EN 1949:2005. In: CEN/TC 216 - Chemical disinfectants and antiseptics, editor. Berlin: Beuth-Verlag. 1–42.

[38] DIN EN ISO 10993–5:2007 D (2007) Biological evaluation of medical devices - Part 5: Tests for in vitro cytotoxicity (lSO/DlS 10993–5:2007);German version prEN ISO 10993-5:2007. In: CEN/TC 206 Biocompatibility of medical and dental materials and devices, editor. Berlin: Beuth-Verlag. 41p.

[39] Edmondson JM, Armstrong LS, Martinez AO (1988) A rapid and simple MTT-based spectrophotometric assay for determining drug sensitivity in monolayer cultures. Journal of Tissue Culture Methods 11: 15–17.

[40] Gómez-Suárez C, Pasma J, van der Borden AJ, Wingender J, Flemming HC, et al (2002) Influence of extracellular polymeric substances on deposition and redeposition of *Pseudomonas aeruginosa* to surfaces. Microbiology 148: 1161–1169. 11932460 10.1099/00221287-148-4-1161

[41] Tielen P, Rosenau F, Wilhelm S, Jaeger KE, Flemming HC, et al (2010) Extracellular enzymes affect biofilm formation of mucoid *Pseudomonas aeruginosa* . Microbiology 156: 2239–2252. 20360178 10.1099/mic.0.037036-0

[42] Sadovskaya I, Vinogradov E, Flahaut S, Kogan G, Jabbouri S (2005) Extracellular carbohydrate-containing polymers of a model biofilm-producing strain, *Staphylococcus epidermidis* RP62A. Infect Immun 73: 3007–3017. 15845508 10.1128/IAI.73.5.3007-3017.2005 PMC1087347

[43] Linton CJ, Sherriff A, Millar MR (1999) Use of a modified Robbins device to directly compare the adhesion of *Staphylococcus epidermidis* RP62A to surfaces. J Appl Microbiol 86: 194–202. 10063617 10.1046/j.1365-2672.1999.00650.x

[44] Rändler C, Matthes R, McBain AJ, Giese B, Fraunholz M, et al (2010) A three-phase in-vitro system for studying *Pseudomonas aeruginosa* adhesion and biofilm formation upon hydrogel contact lenses. BMC Microbiol 10: 282–212. 21062489 10.1186/1471-2180-10-282 PMC2997771

[45] Bowler PG, Duerden BI, Armstrong DG (2001) Wound microbiology and associated approaches to wound management. Clin Microbiol Rev 14: 244–269. 11292638 10.1128/CMR.14.2.244-269.2001 PMC88973

[46] Widerström M, Wiström J, Ek E, Edebro H, Monsen T (2011) Near absence of methicillin-resistance and pronounced genetic diversity among *Staphylococcus epidermidis* isolated from healthy persons in northern Sweden. Apmis 119: 505–512. 21749450 10.1111/j.1600-0463.2011.02757.x

[47] Deredjian A, Colinon C, Brothier E, Favre-Bonté S, Cournoyer B, et al (2010) Antibiotic and metal resistance among hospital and outdoor strains of *Pseudomonas aeruginosa* . Res Microbiol 162: 689–700. 10.1016/j.resmic.2011.06.007 21726631

[48] Hawkey PM (2008) The growing burden of antimicrobial resistance. J Antimicrob Chemother 62: i1–9. 18684701 10.1093/jac/dkn241

[49] Powell DG (1998) Medical Applications of Polycarbonate. Medical Plastics and Biomaterials: http://www.mddionline.com/article/medical-applications-polycarbonate (25.11.2012).

[50] Becker K (1996) Exopolysaccharide production and attachment strength of bacteria and diatoms on substrates with different surface tensions. Microb Ecol 32: 23–33. 8661539 10.1007/BF00170104

[51] Neues Rezeptur-Formularium NRF S.7 (2010) Chlorhexidin zur zahnärztlichen Anwendung. In: Deutscher Arzneimittel-Codex DAC/Neues Rezeptur-Formularium NRF, editor. Eschborn: Govi.

[52] Koban I, Matthes R, Hübner NO, Welk A, Meisel P, et al (2010) Treatment of *Candida albicans* biofilms with low-temperature plasma induced by dielectric barrier discharge and atmospheric pressure plasma jet. New J Phys 12: 073039.

[53] Weltmann K-D, Fricke K, Stieber M, Brandenburg R, Woedtke Tv, et al (2012) New Nonthermal Atmospheric-Pressure Plasma Sources for Decontamination of Human Extremities. IEEE Trans Plasma Sci 40: 2963–2969.

[54] Laroussi M, Richardson JP, Dobbs FC (2002) Effects of nonequilibrium atmospheric pressure plasmas on the heterotrophic pathways of bacteria and on their cell morphology. Appl Phys Lett 81: 772–774.

[55] Pompl R, Jamitzky F, Shimizu T, Steffes B, Bunk W, et al (2009) The effect of low-temperature plasma on bacteria as observed by repeated AFM imaging. New J Phys 11: 115023.

[56] Moore G, Griffith C, Peters A (2000) Bactericidal properties of ozone and its potential application as a terminal disinfectant. J Food Prot 63: 1100–1106. 10945587 10.4315/0362-028x-63.8.1100

[57] Zhang Q, Sun P, Feng HQ, Wang RX, Liang YD, et al (2012) Assessment of the roles of various inactivation agents in an argon-based direct current atmospheric pressure cold plasma jet. J Appl Phys 111: 123305.

[58] Matthes R, Koban I, Bender C, Masur K, Kindel E, et al (2013) Antimicrobial efficacy of an atmospheric pressure plasma jet against biofilms of *Pseudomonas aeruginosa* and *Staphylococcus epidermidis* . Plasma Process Polym 10: 161–166.

[59] Alkawareek MY, Algwari QT, Laverty G, Gorman SP, Graham WG, et al (2012) Eradication of *Pseudomonas aeruginosa* Biofilms by Atmospheric Pressure Non-Thermal Plasma. PLoS One 7: e44289. 22952948 10.1371/journal.pone.0044289 PMC3432087

[60] Cotter JJ, Maguire P, Soberon F, Daniels S, O’Gara JP, et al (2011) Disinfection of meticillin-resistant *Staphylococcus aureus* and *Staphylococcus epidermidis* biofilms using a remote non-thermal gas plasma. J Hosp Infect 78: 204–207. 21601949 10.1016/j.jhin.2011.03.019

[61] Kamgang JO, Briandet R, Herry JM, Brisset JL, Naitali M (2007) Destruction of planktonic, adherent and biofilm cells of *Staphylococcus epidermidis* using a gliding discharge in humid air. Journal of applied microbiology 103: 621–628. 17714395 10.1111/j.1365-2672.2007.03286.x

[62] Dobrynin D, Fridman G, Friedman G, Fridman A (2009) Physical and biological mechanisms of direct plasma interaction with living tissue. New J Phys 11: 115020.

[63] Montie TC, Kelly-Wintenberg K, Roth JR (2000) An overview of research using the one atmosphere uniform glow discharge plasma (OAUGDP) for sterilization of surfaces and materials. IEEE Trans Plasma Sci 28: 41–50.

[64] Matthes R, Bekeschus S, Bender C, Koban I, Hübner NO, et al (2012a) Pilot-study on the influence of carrier gas and plasma application (open resp. delimited) modifications on physical plasma and its antimicrobial effect against *Pseudomonas aeruginosa* and *Staphylococcus aureus* . GMS Krankenhhyg Interdiszip 7: Doc02. 22558036 10.3205/dgkh000186 PMC3334954

[65] Hähnel M, Diener A, Kolukisaoglu U, Weltmann KD, Thurow K (2011) The Influence on Cell Growth Properties in Different Microtiterplate Types by Corona-Dielectric Barrier Discharge Plasma at Atmospheric Pressure. Plasma Process Polym 8: 70–76.

[66] Isbary G, Heinlin J, Shimizu T, Zimmermann JL, Morfill G, et al (2012) Successful and safe use of 2 min cold atmospheric argon plasma in chronic wounds: results of a randomized controlled trial. Br J Dermatol 167: 404–410. 22385038 10.1111/j.1365-2133.2012.10923.x PMC7161860

[67] Metelmann H-R, Woedtke Tv, Bussiahn R, Weltmann K-D, Rieck M, et al (2012) Experimental Recovery of CO_2_-Laser Skin Lesions by Plasma Stimulation. American Journal of Cosmetic Surgery 29: 52–56.

[68] Emmert S, Brehmer F, Hänßle H, Helmke A, Mertens N, et al (2013) Atmospheric pressure plasma in dermatology: Ulcus treatment and much more. Clinical Plasma Medicine 1: 24–29.

